# Microbial-enzyme synergistic treatment stabilizes surface microbial communities and enhances flavor quality during tobacco leaf aging

**DOI:** 10.1186/s40643-026-01052-1

**Published:** 2026-04-15

**Authors:** Chunping Xu, Yizhe Sun, Yuntao Fan, Qu Lili, Xiao Zhang, Meizhou Ding, Ma Rong

**Affiliations:** 1 College of Tobacco Science and Engineering, Zhengzhou University of Light Industry, Zhengzhou, 450000 China; 2 Research Center, Hebei Tobacco Industry Co., Ltd., Shijiazhuang, 052165 China; 3 Henan High-Tech Industry Co., Ltd., Henan Academy of Sciences, Zhengzhou, 450000 China; 4 Research Center, Henan Tobacco Industry Co., Ltd., Zhengzhou, 450000 China

**Keywords:** Tobacco aging, Microbial community, Volatile compounds, Microbial-enzyme co-fermentation

## Abstract

Tobacco aging is a critical for developing desirable flavor profiles, driven primarily by microbial and enzymatic activities. This study systematically evaluated the effects of *Bacillus clausii* inoculation (FJ) and *B. clausii*-cellulase co-treatment (JM) on the surface microbial communities and aroma compounds of Yunyan 87 tobacco leaves during 9 months of aging, with natural aging as the control (CK). High-throughput 16S rRNA sequencing revealed that the JM treatment significantly increased microbial diversity and enhanced the structural similarity of microbial communities across different aging stages compared with the CK and FJ treatments, while also promoting the proliferation of beneficial taxa (e.g., *Bacteroidota*, *Cyanobacteria*). PICRUSt functional prediction revealed that JM enriched metabolic pathways related to carbohydrate metabolism (18.2–20.6%), amino acid metabolism (15.1–16.7%), and metabolism of cofactors and vitamins (16.5–17.7%)–key pathways for flavor precursor conversion. A total of 29 key aroma compounds (odor activity value > 1) were significantly influenced the quality of tobacco leaves, with JM significantly increasing total volatile content (317.1–388.8 μg/g vs. 124.7–143.0 μg/g in CK and 283.2–300.8 μg/g in FJ). Notably, JM promoted the accumulation of esters (ethyl palmitate, ethyl linoleate) and ketones (4,7,9-megastigmatrien-3-one, damascenone), contributing desirable fruity, floral, and sweet notes. Correlation analysis linked *Pseudomonas*, *Bacillus*, and *Acinetobacter* to the formation of key volatiles. These findings demonstrate that microbial-enzyme co-fermentation enriched desirable aromas, and stabilized microbial communities, providing a practical and efficient strategy for industrial production of high-quality aged tobacco.

**Graphical abstract:**

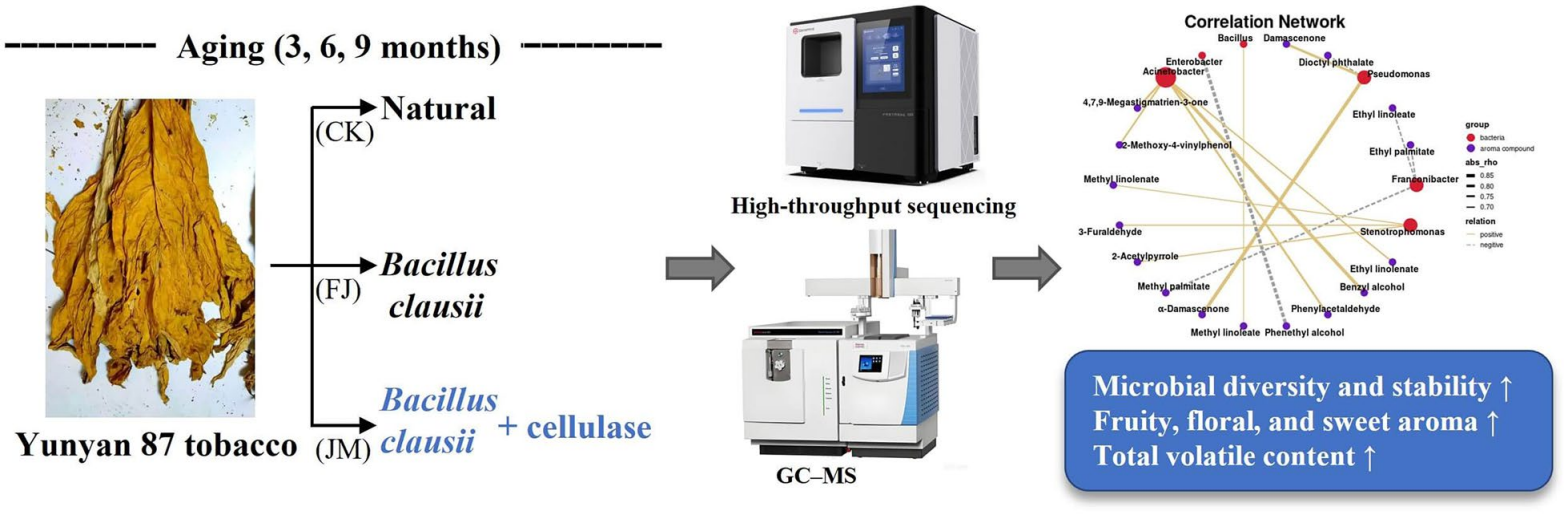

**Supplementary Information:**

The online version contains supplementary material available at 10.1186/s40643-026-01052-1.

## Introduction

Tobacco is among the most extensively cultivated commercial crops worldwide, and China is one of the largest producers and consumers, accounting for approximately 35% of global production and nearly 32% of total sales (Shan et al. 2025). The quality of tobacco leaves is largely influenced not only by cultivation conditions, such as climate, humidity, and altitude (Hu et al. 2022), but also by post-harvest aging and fermentation processes, which play a decisive role in shaping aroma quality and sensory characteristics (Li et al. 2020). During aging, complex biochemical and microbial interactions occur, leading to the degradation of undesirable compounds and the formation of desirable aroma substances (Li et al. 2020). These processes involve the conversion of macromolecules such as starch, proteins, and cellulose into low-molecular-weight flavor precursors through microbial metabolism and enzymatic catalysis (Mai et al. 2025). However, traditional natural aging relies on indigenous microorganisms and ambient environmental conditions, often resulting in long processing times and inconsistent product quality (Liu et al. 2021; Zheng et al. 2022). Consequently, strategies to accelerate aging and improve flavor quality consistency have attracted increasing attention.

The application of exogenous microorganisms and enzyme preparations to accelerate tobacco aging and enhance flavor quality has therefore been widely explored (Pei et al. 2025; Zhang et al. 2023). Among them, *Bacillus* species are widely used due to their robust extracellular enzymatic activities, including cellulase, amylase, and protease production, which facilitate the degradation of structural and storage compounds in tobacco leaves (Wei et al. 2024). In parallel, commercial enzymes like cellulase have been applied to specifically hydrolyze cellulose and hemicellulose, thereby releasing fermentable sugars and bound aroma precursors and improving substrate accessibility (Hao et al. 2025). The combined application of microorganisms and enzymes has been proposed as a synergistic strategy in which enzymatic hydrolysis enhances substrate availability, while microbial metabolism further converts released sugars, amino acids, and lipid intermediates into volatile aroma compounds (Shu et al. 2026). Nevertheless, most existing studies have primarily focused on compositional changes in volatiles and succession of microbial community. While microbial-enzyme co-fermentation shows promise in modulating community structure and flavor-related metabolism, the extent to which such strategies coordinately shape microbial functional potential and drive flavor formation during tobacco aging remains poorly resolved, especially with regard to underlying mechanisms.

The surface microbiota of tobacco leaves play a central role in determining the outcomes of the aging process and final product quality (Wang et al. 2022, 2024b). High-throughput sequencing technologies, particularly 16S rRNA gene amplicon sequencing, have enabled comprehensive characterization of microbial community composition and functional potential (Di Bella et al. 2013). Previous studies have demonstrated that microbial inoculation can significantly alter the abundance and diversity of dominant genera such as *Bacillus*, *Acinetobacter*, *Staphylococcus*, and *Aspergillus*, and modulate metabolic pathways associated with carbohydrate, amino acid, and lipid metabolism (Yin et al. 2025). Microbial metabolism also directly influences the transformation of flavor precursors into odor-active compounds. For example, polysaccharide and protein degradation can enhance Maillard reaction products and Strecker aldehydes (Jiang et al. 2024; Zhu et al. 2025), while carotenoid and lipid metabolism contributes to the generation of alcohols, ketones, and esters (Mai et al. 2024). However, the correlative and functional relationships between microbial community dynamics and aroma compound formation, particularly under microbial–enzyme co-treatment conditions, have yet to be systematically demonstrated, leaving the mechanistic links between microbial-enzyme interactions and flavor development incompletely understood.

Therefore, this study investigated the effects of bacterial inoculation and bacterial-enzyme co-fermentation on the microbial community structure and flavor formation of tobacco leaves during aging. By integrating high-throughput sequencing with gas chromatography–mass spectrometry (GC–MS), the effects of different aging methods on surface microbial succession, metabolic function, and aroma compound profiles were analyzed. Furthermore, correlations between the microbial community and key aroma compounds were elucidated to provide potential mechanistic insights into microbial-enzyme synergistic effects. This work aims to provide a theoretical insight and practical guidance for the industrial application of microbial-enzyme co-fermentation to achieve consistent and high-quality tobacco aging.

## Materials and methods

### Materials and chemicals

Yunyan 87 tobacco leaves, utilized for the aging process, were provided by Guangxi Tobacco Industry Co., Ltd. *B. clausii* was isolated and screened by the Tobacco Biotechnology Research Laboratory of Zhengzhou University of Light Industry. The strain was selected from tobacco leaf, deposited in CCTCC (CCTCC AB 2025104), and identified as *B. clausii* by full-length 16S rRNA sequencing and NCBI alignment. The enzymatic agent, cellulase (50 U/mg, solid formulation), was sourced from Shanghai yuanye Bio-Technology Co., Ltd. Chromatographic grade dichloromethane was provided by Thermo Fisher Scientific (China) Co., Ltd. The internal standard phenyl ethyl acetate was purchased from Shanghai Aladdin Biochemical Technology Co., Ltd. And all other chemicals used in this study were analytical grade.

### Preparation of strain

The *B. clausii* strain used in this study, which had been previously isolated and screened from tobacco leaf surfaces for its demonstrated efficacy in improving tobacco sensory quality, was inoculated onto LB solid medium using a sterile loop in a laminar flow hood. The inoculated plates were subsequently incubated in a constant-temperature incubator at 37 °C for 24 h.

Suspension preparation: Two loops of *B. clausii* from the solid medium were inoculated into LB liquid medium and cultured at 37 °C and 180 r/min for 48 h. The resulting seed culture was centrifuged at 6000 r/min for 10 min, after which the supernatant was discarded. The pellet was resuspended in sterile water, and the optical density was measured using a UV spectrophotometer. The suspension was diluted to an OD_600_ of 1.45, corresponding to a bacterial concentration of 10^8^ CFU/mL. This bacterial suspension was set aside for further use.

Bacterial-enzyme mixture preparation: 1 g of cellulase (with a dosage of 50 U per gram of tobacco leaves) was accurately weighed and dissolved in 80 mL of the prepared bacterial suspension. The mixture was stirred uniformly to form the bacterial-enzyme mixture.

### Pre-treatment of tobacco leaves before aging

The prepared bacterial suspension and bacterial-enzyme mixture were uniformly sprayed onto the surface of the tobacco leaves at a dosage of 80 mL per 1000 g of tobacco. An equal volume of sterile water was applied to the control treatment. After treatment, the tobacco leaves were transferred to an aging warehouse and subjected to fermentation.

A total of 13.5 kg of tobacco leaves were used in this study and randomly assigned to three treatment groups: control (CK), bacterial suspension (FJ), and bacterial-enzyme mixture (JM). Each group contained 0.5 kg of tobacco per independent fermentation batch, and all treatments were performed in triplicate under identical environmental conditions (temperature: 25–30 °C; humidity: 60–65%). Samples sprayed with sterile water and aged for 3, 6, and 9 months were labeled as CK-3, CK-6, and CK-9, respectively. Correspondingly, samples treated with the bacterial suspension were designated as FJ-3, FJ-6, and FJ-9, while those treated with the bacterial-enzyme mixture were denoted JM-3, JM-6, and JM-9. At the end of the designated aging period, the entire 0.5 kg of tobacco leaves were collected and thoroughly homogenized to ensure sample homogeneity. The homogenized sample was then split into two portions: 400 g were stored at −20 °C for volatile compound analysis and 100 g were ground in liquid nitrogen and stored at −80 °C for microbial DNA extraction. For clarity, samples collected at 3, 6, and 9 months were defined as representing the early, middle, and late stages of fermentation, respectively.

### DNA extraction, amplification and sequencing of microbiota

The nine samples subjected to different aging methods were aseptically cut into fine pieces. Total microbial DNA was extracted from the tobacco leaves following the instructions of the E.Z.N.A.™ Mag-Bind Soil DNA Kit according to a previously published method with minor modifications (Zhang et al. 2020). Using the total DNA of the aforementioned samples as the template, the first round of PCR amplification was performed. Bacterial 16S rRNA sequences were amplified using the primers 5′-CCT ACG GRR BGC ASC AGK VRV GAA/T3′ and 5′-GGA CTA CNV GGG TWT CTA ATC C-3′. The reaction mixture consisted of: 10 ng of DNA template, 15 µL of 2 × Hieff® Robust PCR Master Mix, 1 µL of Bar-PCR primer F, 1 µL of Primer R, and ddH₂O added to a final volume of 30 µL. The amplification protocol was as follows: initial denaturation at 94 °C for 3 min; 25 cycles of denaturation at 94 °C for 30 s, annealing at 55 °C for 20 s, and extension at 72 °C for 30 s; followed by a final extension at 72 °C for 5 min. A second round of PCR amplification was conducted using the products from the first PCR as templates, with the introduction of Illumina bridge-PCR compatible primers. The reaction mixture and thermal cycling conditions were identical to those used in the first round. The PCR products were examined by electrophoresis on a 2% (*w/v*) agarose gel. The targeted 16S rRNA gene fragments were purified using a multifunctional DNA purification and recovery kit. The purified PCR products were subsequently sent to Sangon Biotech (Shanghai) Co., Ltd. for bacterial 16S rRNA sequencing on the MiSeq high-throughput sequencing platform.

The raw sequences were initially processed using Cutadapt to remove primer and adapter sequences. Paired-end reads were then assembled into single sequences based on their overlap regions. Subsequently, the sequences were demultiplexed and assigned to corresponding samples according to their barcode tags. The processed sequences for each sample were further refined using Prinseq to obtain high-quality effective sequences. The sequences were clustered into operational taxonomic units (OTUs) using a 97% identity cutoff. Subsequently, chimeric sequences were identified and filtered out from the dataset. The representative sequences of OTUs were taxonomically annotated. Microbial community diversity indices for the 9 tobacco leaf samples were calculated using Mothur version 1.31.2. Principal coordinates analysis (PCoA) was performed to examine differences in OTU distribution among tobacco samples subjected to different aging methods. The relative abundances of microbial communities at the phylum and genus levels across all samples were visualized and analyzed using Origin 2022. To predict the functional profiles of the microbial communities in different tobacco samples, PICRUSt was employed. The accuracy of functional prediction was evaluated using the weighted nearest sequenced taxon index (Weighted NSTI), which represents the weighted average distance between the sequences in each sample and the reference genomes in the database.

### Detection of volatile compounds

The content of aroma components in tobacco leaves was analyzed using GC–MS coupled with simultaneous distillation–extraction (SDE) for sample preparation as previously described method (Wu et al. 2025). Tobacco leaf samples were dried at 60 °C and ground into a fine powder using a 60-mesh sieve. Exactly 25.00 g of the powdered sample was placed into a 1000 mL round-bottom flask, mixed with 20 g of NaCl and 400 mL of deionized water, and shaken thoroughly. The mixture was then connected to one end of the SDE apparatus and heated in a 60 °C water bath for 150 min. The extraction solvent was dichloromethane. After the extraction was completed, 50 μL of 0.871 mg/mL phenyl ethyl acetate solution was added as the internal standard. After thorough shaking and mixing, anhydrous sodium sulfate was added for drying treatment. Subsequently, the sample was concentrated to approximately 1 mL, filtered through a 0.45 μm membrane, and subjected to GC–MS analysis.

GC–MS was performed using an Agilent 7890A-5975C system equipped with an HP-5MS capillary column (30 m × 0.25 mm × 0.25 μm; Agilent 19091S-433). The oven temperature program was set as follows: initial temperature 60 °C, ramped at 2 °C/min to 260 °C and held for 10 min, then increased to 280 °C at 5 °C/min. The MS ion source and quadrupole temperatures were maintained at 230 °C and 150 °C, respectively, with a mass scan range of 30–550 m/z. Compound identification was carried out using the NIST20 mass spectral library, and data processing was performed with Agilent MassHunter Workstation software. The concentration of volatile compound was calculated using the following equation:

$$ {\mathrm{Content}} ({\mu g}/{\mathrm{g}}) = C_{i} \times v_{i} \times A_{s} /m_{0} \times A_{i} $$

where $$ C_{i}$$ is the concentration of internal standard, mg/mL; $$v_{i}$$ is the volume of internal standard, μL; $$A_{s}$$ and $$ A_{i}$$ are peak area of volatile compound and the internal standard, respectively; $$m_{0}$$ refers to the quantity of the sample, g.

The odor activity value (OAV) is determined by dividing the concentration of volatile compound by its corresponding odor threshold (Wu et al. 2025).

### Statistical analysis

All experiments were conducted in triplicate, and the results were expressed as mean ± standard deviation (SD). Statistical analysis was performed using a one-way analysis of variance (ANOVA), followed by Duncan’s significance test using SPSS 19.0, with statistical significance defined as *p* < 0.05. Bar charts were generated using Origin 2021 (OriginLab, Northampton, MA, USA). Partial least squares discriminant analysis (PLS-DA) was carried out with SIMCA 14.1 (Umetrics, Umeå, Sweden). PCoA and Spearman correlation coefficients were performed using the Chiplot online tool (https://www.chiplot.online). Significant correlations were screened using a threshold of |ρ|≥ 0.7 and an FDR-adjusted *p* < 0.05. Correlation networks were conducted with Cytoscape (v. 3.10.1).

## Results and discussion

### Microbiological analysis in tobacco leaves with different aging treatments

#### Overview of the microbial community

The diversity of bacterial communities was evaluated by high-throughput sequencing of the 16S rRNA variable region. As shown in Fig. S1, rarefaction curves approached stabilization with increasing sequencing depth, indicating that the majority of microbial diversity within the samples had been captured. The coverage indices were all higher than 99.8%, further confirming that the sequencing effort was sufficient to represent the bacterial diversity and support reliable taxonomic classification. After filtering out singletons, a total of 81,103–94,247, 78,697–92,448, and 70,182–87,463 high-quality bacterial sequences in CK, FJ, and JM samples, respectively, were retained for subsequent analysis (Table S1). These sequences were clustered into OUT at a 97% similarity threshold.

#### α- and β-diversity

**Fig. 1:**
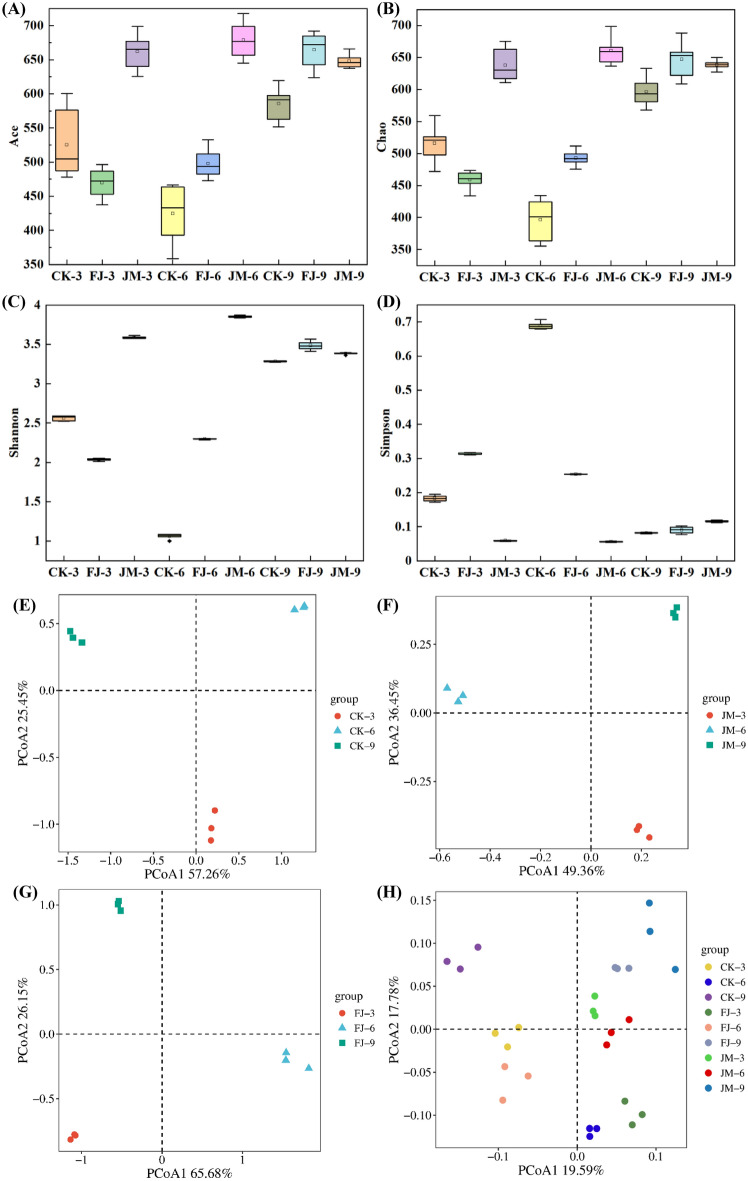
Microbial community diversity and composition in tobacco leaves with different aging treatments. **A** Ace index; **B** Chao index; **C** Shannon index; **D** Simpson diversity index; **E**–**H** PCoA analysis based on Bray–Curtis distance. CK: sterile water control; FJ: bacterial treatment; JM: bacterial-enzyme treatment

To evaluate the richness and diversity of microbial communities during tobacco aging, α-diversity indices—including Ace, Chao, Shannon, and Simpson—were analyzed (Ruan et al. 2021). The Ace and Chao indices reflect species richness, whereas the Simpson diversity and Shannon indices indicate species diversity. Higher values of the Simpson diversity index indicate greater microbial diversity. In contrast, lower Shannon index values reflect reduced microbial diversity. As shown in Fig. 1, the Ace and Chao indices revealed significantly higher bacterial richness in JM samples compared to FJ samples at 3 and 6 months of fermentation. However, this trend reversed at 9 months (Fig. 1A and B). The Shannon index was significantly higher in the JM samples compared with the CK and FJ samples, suggesting that the JM treatment supported a more diverse distributed microbial community (Fig. 1C and D). Throughout the fermentation process, the richness of FJ samples gradually increased, whereas JM samples showed minor change. Microbial diversity increased progressively in the FJ samples but decreased in the JM samples. These results indicated that the richness and diversity of microbial communities varied with the aging time of tobacco leaves. Additionally, the application of a bacterial suspension and enzyme mixture significantly enhanced both richness and diversity compared to natural aging alone, with exception of the nine-month time point. These findings align with previous studies demonstrating that exogenous microbial inoculation can substantially reshape the microbial community structure in tobacco leaves (Shu et al. 2023).

β-diversity was analyzed to characterize structural differences in microbial community composition among different aging treatments and temporal stages. Principal coordinate analysis (PCoA) based on Bray–Curtis distance was performed to evaluate the variation and similarity of microbial community structure among samples (Fig. 1E–G), which is a classic approach for β-diversity characterization in tobacco fermentation research (Wu et al. 2024). The first two principal coordinates (PCo1 and PCo2) explained 82.71%, 85.81%, and 91.83% of the total variance in CK, JM, and FJ treatments, respectively, indicating strong explanatory power of the PCoA ordination for the observed microbial community structural variation. Across all treatments, samples from different aging time points were clearly separated based on the x-axis (PCo1) and y-axis (PCo2), with the most distinction observed along the x-axis, which reflected a pronounced temporal succession pattern of the microbial community structure during aging. Notably, further comparison of β-diversity patterns via PCoA (Fig. 1H) showed that JM treatments exhibited tighter clustering across the three aging stages compared to those from CK or FJ treatments, indicating that the JM treatment promoted grater stability in microbial community composition over time. Moreover, clear separation among samples (CK, FJ, and JM) was observed, suggesting a pronounced differentiation of the microbial community. The results are consistent with previous studies that PCoA was successfully used to distinguish microbial community differences among different treatment groups or fermentation stages (Li et al. 2024; Wu et al. 2024). For instance, Zhang et al. (2024b) employed PCoA to analyze microbial community variations during cigar tobacco leaves fermentation, and the results clearly clustered bacterial and fungal communities into three distinct groups. Collectively, these results demonstrated that aging time and treatment type are key factors shaping microbial community structure, with the JM treatment significantly enhancing community homogeneity and driving a distinct succession pattern compared to CK and FJ treatments.

#### Dynamic changes in microbial communities

**Fig. 2:**
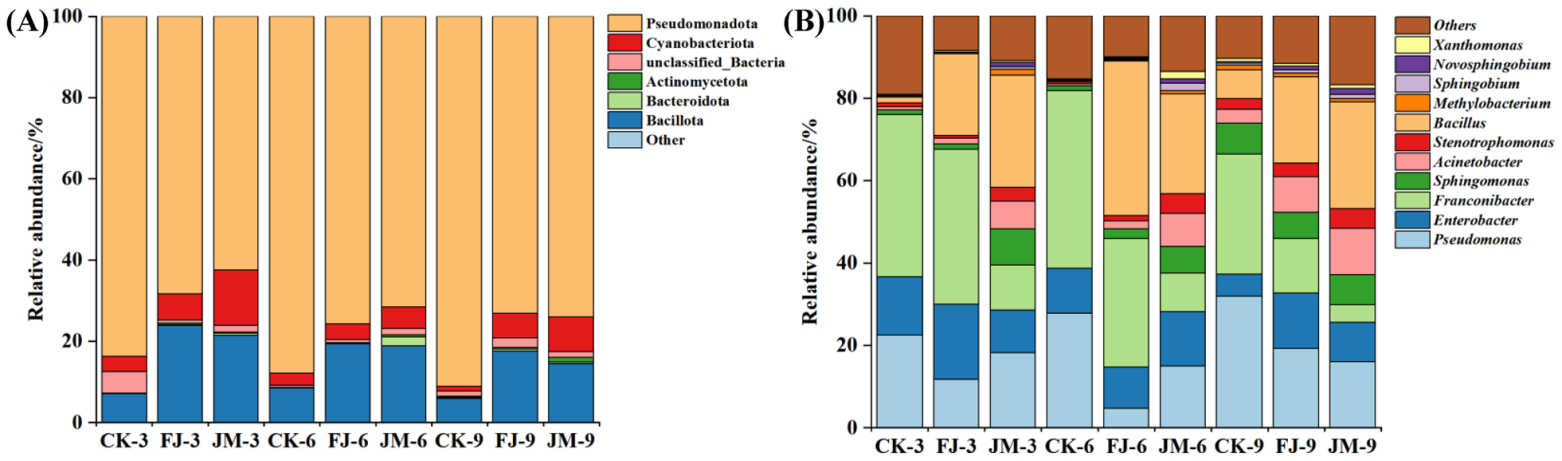
Dynamic changes in the relative abundance of microbial communities at the phylum (**A**) and genus (**B**) levels in tobacco leaves with different aging treatments. CK: sterile water control; FJ: bacterial treatment; JM: bacterial-enzyme treatment

The relative abundance of the main microbial community (with a relative abundance > 1%) in tobacco leaves with different aging treatments was analyzed at phylum and genus levels (Fig. 2). At the phylum level (Fig. 2A), Pseudomonadota and Bacillota were the dominant taxa across all treatments, collectively accounting for 80–97% of the total bacterial community. Compared to Ck, FJ treatment increased the relative abundance of Bacillota (by 12.3–18.7% across aging stages), while JM treatment significantly promoted the proliferation of Bacteroidota and Cyanobacteria (relative abundance increased by 5.8–9.2% and 3.1–4.5%, respectively), while maintaining high levels of Pseudomonadota and Bacillota (Fig. 2A). Temporally, Pseudomonadota and Actinomycetota increased with aging time in JM, whereas Bacillota declined; this shift may reflect enhanced metabolic cooperation between these phyla during flavor precursor conversion.

At the genus level (Fig. 2B), the predominant bacterial communities in tobacco leaves varied among treatments. In the CK treatment, the dominant genera were *Franconibacter* (29.11–39.29%), *Pseudomonas* (22.50–31.88%), and *Enterobacter* (5.40–14.16%), which collectively accounted for 61.80–70.80% of the total community across aging stages. In contrast, the FJ treatment was dominated by *Franconibacter* (13.29–37.63%), *Bacillus* (19.78–37.41%), *Enterobacter* (10.09–18.13%), and *Pseudomonas* (4.61–19.22%), while the JM treatment exhibited a distinct profile dominated by *Bacillus* (24.25–27.30%), *Pseudomonas* (15.00–18.16%), *Enterobacter* (9.46–13.12%), and *Franconibacter* (4.33–10.99%), which showed a more balanced genus distribution. While the dominant bacterial genera (e.g., *Franconibacter*, *Pseudomonas*, *Enterobacter*, *Bacillus*) were conserved across treatments, their relative abundances shifted substantially. These quantitative shifts, along with changes in the relative abundance of minor taxa, collectively indicated that the application of microbial-enzyme co-fermentation significantly altered the microbial community structure and enhanced microbial diversity. Over the aging period, temporal shifts in genus abundance were also observed. In the CK group, the relative abundance of *Pseudomonas* and *Sphingomonas* gradually increased during aging, whereas that of *Enterobacter* decreased. Notably, *Pseudomonas* and *Franconibacter* together accounted for 61.79%, 70.78%, and 60.99% of the microbiota at 3, 6, and 9 months, respectively, indicating that these two genera consistently represented major components of the community under control conditions. In the FJ treatment, several genera, including *Sphingomonas*, *Acinetobacter*, *Stenotrophomonas*, *Methylobacterium*, *Sphingobium*, and *Novosphingobium*, exhibited increasing trends over time, whereas *Franconibacter* declined. Meanwhile, the combined relative abundances of *Pseudomonas*, *Franconibacter*, and *Bacillus* reached 69.17%, 73.26%, and 53.48% at 3, 6, and 9 months, respectively, suggesting that these genera constituted major and relatively stable components of the bacterial community in this treatment. Similarly, in the JM treatment, the relative abundance of *Acinetobacter* and *Novosphingobium* increased with aging time, whereas that of *Franconibacter* decreased. The combined relative abundance of *Pseudomonas* and *Bacillus* constituted 45.46%, 39.25%, and 42.05% of the community at 3, 6, and 9 months, indicating that these genera also remained among the major components of the microbial community under JM treatment.

#### Biomarker microorganisms in different aging treatments

**Fig. 3:**
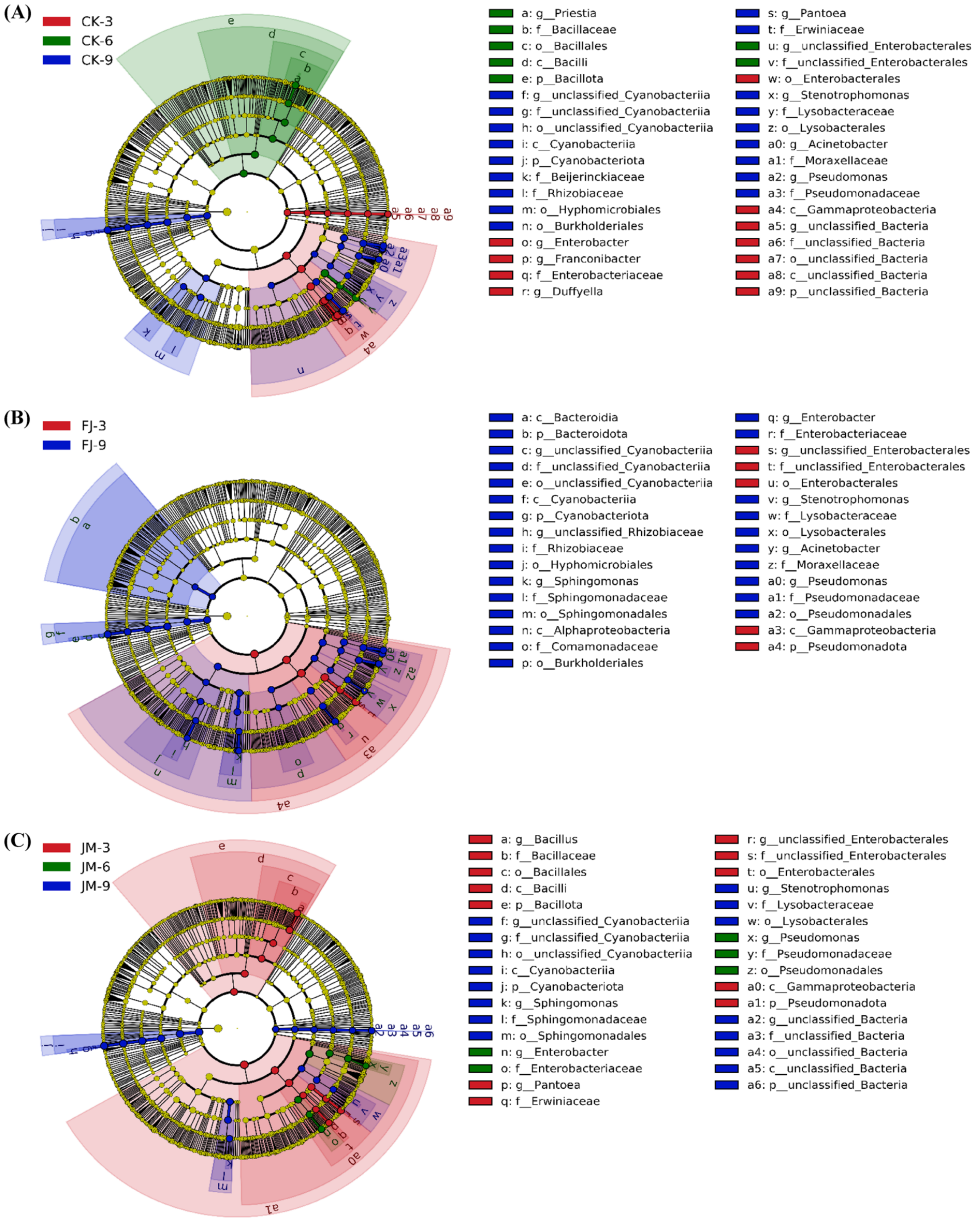
LEfSe analysis of microbial communities in tobacco leaves with different aging treatments. **A** CK; **B** FJ; **C** JM. CK: sterile water control; FJ: bacterial treatment; JM: bacterial-enzyme treatment

Linear discriminant analysis (LDA) effect size (LEfSe) is an effective approach for identifying taxa that exhibit statistically significant and biologically meaningful differences across groups, particularly during dynamic process such as fermentation (Zhang et al. 2025c). This approach provides valuable insights into microbial succession and their potential associations with final product quality. In this study, LEfSe was employed to identify differentially abundant bacterial taxa in the CK, FJ, and JM groups across three aging time points (3, 6, and 9 months). The evolutionary cladograms (Fig. 3A–C) illustrate the phylogenetic distribution of identified biomarkers from phylum to species level, radiating from the inner to outer circles. Complementary biomarker (LDA > 4.0, *p* < 0.05) distributions for each treatment and aging points were shown in supplementary Fig. S2A–C. Specifically, in the CK treatments (Fig. 3A), the microbial composition at 3 months was characterized by biomarkers such as *Franconibacter* and *Enterobacter*. By 6 months, the community shifted towards taxa within *Enterobacterales* and *Priestia*. At 9 months, a further succession was observed, with significant enrichment of *Pseudomonas*, *Pantoea*, *Acinetobacter*, and *Stenotrophomonas*. In the FJ treatments (Fig. 3B), only *Enterobacterales* was significantly enriched at 3 months. By 9 months, biomarker diversity increased substantially, including *Pseudomonas*, *Enterobacter*, *Acinetobacter*, *Sphingomonas*, and *Stenotrophomonas*. No microbial taxa met the significance criteria (LDA > 4.0, *p* < 0.05) at the 6-month time point and therefore no biomarkers were identified for FJ-6. This suggested that, within the FJ treatment, the microbial community at 6 months was not sufficiently distinct from those at 3 and 9 months to produce differential biomarkers under these parameters. In contrast, the JM treatment exhibited a clearer temporal succession of biomarkers across aging period (Fig. 3C). At 3 months, key biomarkers included *Enterobacterales*, *Bacillus*, and *Pantoea*. At 6 months, the community became less complex, with only *Pseudomonas* and *Enterobacter* identified as biomarkers. By 9 months, the biomarker profile diversified again to include *Cyanobacteriia*, *Sphingomonas*, and *Stenotrophomonas*. Overall, LEfSe analysis indicated that both aging treatment and duration were associated with successional dynamics of the microbial communities. The observed temporal progression of characteristic microorganisms suggested a potential linkage to the divergence in flavor quality among tobacco leaves aged under different conditions.

#### Microbial function prediction analysis

**Fig. 4:**
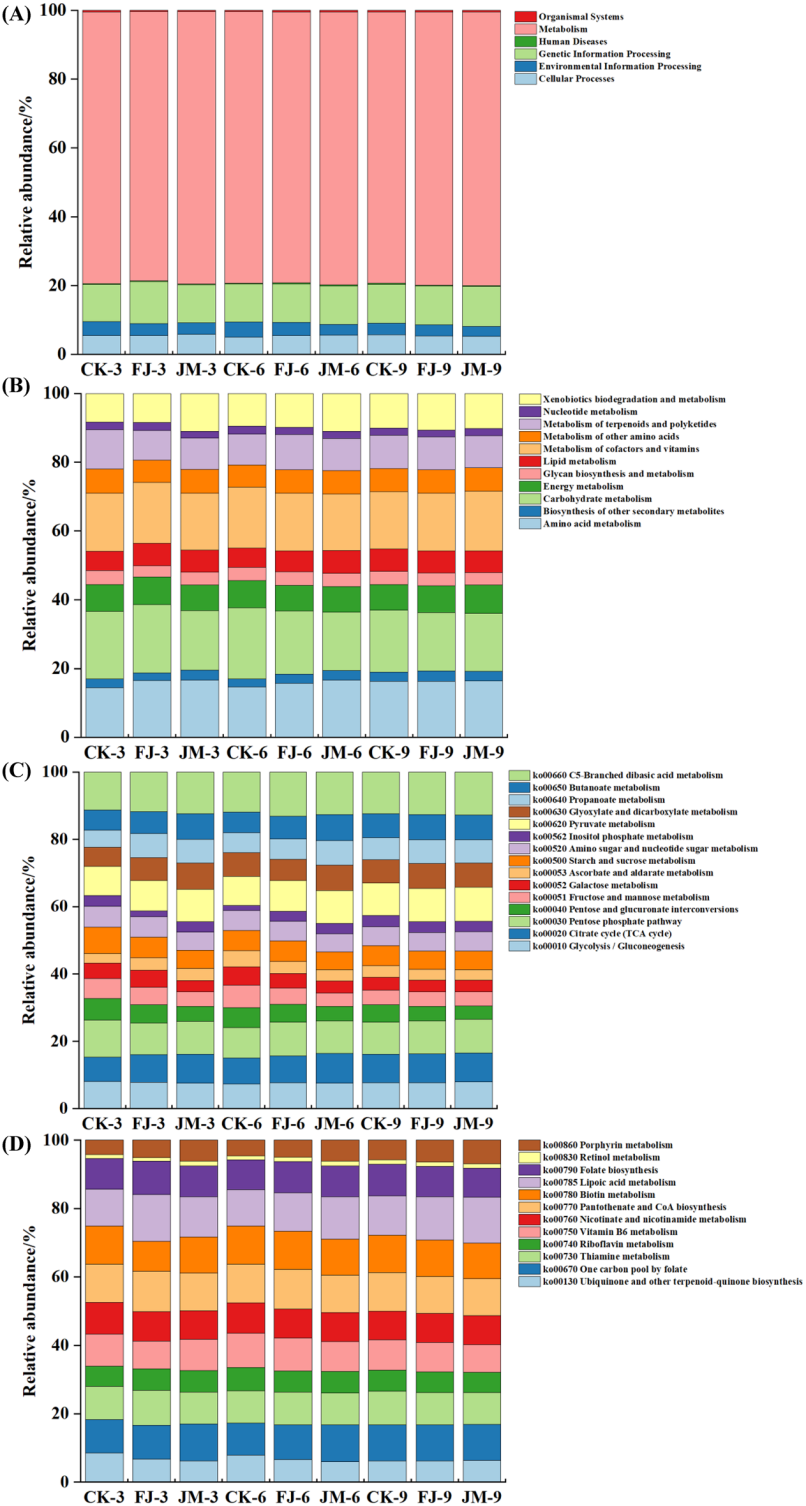
KEGG function prediction. **A** (Level 1) metabolic pathways; **B** (Level 2) metabolism-related pathways; **C** (Level 3) carbohydrate metabolism; **D** (Level 3) amino acid metabolism. CK: sterile water control; FJ: bacterial treatment; JM: bacterial-enzyme treatment

High-throughput sequencing data were aligned against the KEGG database, and functional potentials of the microbial communities in tobacco leaves were predicted using PICRUSt. As shown in Fig. 4A, six major categories were identified at KEGG Level 1 across all aging treatments, including Cellular Processes, Environmental Information Processing, Genetic Information Processing, Metabolism, Organismal Systems, and Human Diseases. Among these, metabolic pathways were predominant, accounting for 78.22% to 79.57% of the predicted functional profiles. Minor temporal shifts in metabolic functions were observed among the microbial communities. For instance, under the same aging duration, the JM treatment exhibited higher metabolic activity compared to the CK and FJ treatments. Furthermore, within the JM treatment, the relative abundance of genes associated with metabolic functions progressively increased with extended aging time. This enrichment of metabolism-related genes, particularly those involved in amino acid, carbohydrate, and lipid metabolism, suggested enhanced microbial-driven biochemical transformations during aging, which have been widely reported to facilitate precursor degradation and promote aroma compound formation in fermented tobacco (Cai et al. 2025; Mai et al. 2025).

Further classification within metabolism-related pathways identified a total of 11 metabolic pathways at KEGG Level 2 (Fig. 4B). Among these, carbohydrate metabolism pathway was the most abundant, accounting for 16.72–20.60%, followed by metabolism of cofactors and vitamins and amino acid metabolism, which accounting for 16.49–17.69% and 14.48%–16.67%, respectively. These results suggested that carbohydrate metabolism, metabolism of cofactors and vitamins, and amino acid metabolism pathways were dominant during aging and likely contribute substantially to volatile compound formation of tobacco leaves. These findings align with previous studies on cigar, which also reported high relative abundances of carbohydrate metabolism, amino acid metabolism, energy metabolism, cofactor and vitamin metabolism, and membrane transport among the most active functional pathways (Pan et al. 2025). In carbohydrate metabolism (Fig. 4C), 15 pathways were detected, with branched dibasic acid metabolism accounted for the highest proportion (11.35–13.14%), followed by pentose phosphate pathway (9.02–10.96%) and pyruvate metabolism pathway (8.65–10.20%). The enrichment of these pathways suggested an active conversion of carbohydrates into central metabolic intermediates, providing both energy and precursor metabolites for downstream biosynthetic processes. In particular, the pentose phosphate pathway supplies reducing power in the form of NADPH and ribose-5-phosphate for anabolic reactions, whereas pyruvate metabolism serves as a key metabolic hub linking carbohydrate degradation with amino acid, fatty acid, and secondary metabolite biosynthesis (Ren et al. 2025). In metabolism of cofactors and vitamins (Fig. 4D), a total of 12 metabolic pathways were identified, with lipoic acid metabolism was the most prominent (10.50–13.67%), followed by pantothenate and CoA biosynthesis (10.77–11.84%) and biotin metabolism (8.74–11.24%). Generally, metabolism of cofactors and vitamins constitutes an important regulatory layer of cellular metabolism, as these compounds are required to maintain normal physiological activity and to support enzyme-catalyzed reactions (Zhang et al. 2024a). The relatively high representation of these pathways in the present study was thus indicative of a metabolically active microbial system that was capable of sustaining multiple downstream metabolic processes, including carbohydrate, amino acid, lipid, and energy metabolism.

### Changes of volatile compounds in tobacco leaves with different aging treatments

**Fig. 5:**
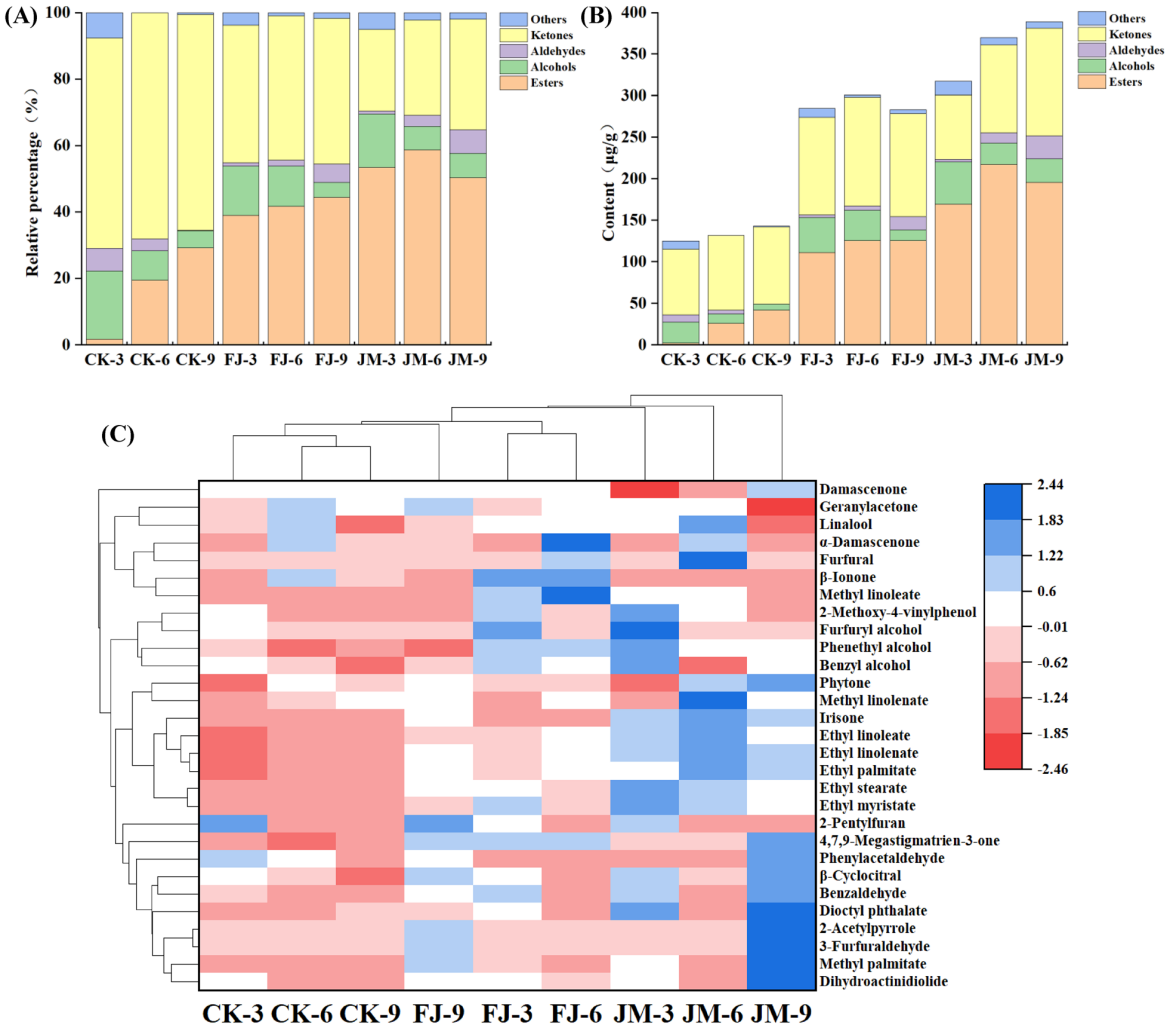
Aroma profiles in tobacco leaves with different aging treatments. **A** The relative percentage and (**B**) average content of aroma compounds in each category. **C** Concentration cluster heatmap of aroma compounds across samples. CK: sterile water control; FJ: bacterial treatment; JM: bacterial-enzyme treatment

A total of 326 volatile compounds were detected by GC–MS, among which 29 substances were the main aromatic compounds of aged tobacco leaves, including 10 esters, 4 alcohols, 5 aldehydes, 7 ketones, and 3 others (Table 1). As shown in Fig. 5A, ketones consistently constituted the most abundant class of volatiles in CK and FJ treatments, followed by esters and alcohols. While in JM treatment, esters were the most abundant aroma compounds, followed by ketones and alcohols. Among all the treatments, aldehydes accounted for less than 7.5% of the total. As aging progressed, the relative abundance of ketones and esters continued to rise, while that of alcohols declined. The observed compositional shift suggested that a potential transformation of aroma compounds during the aging process. Ketones and esters are known for their low odor thresholds and desirable aromatic properties, such as sweet, fruity, and floral notes. Therefore, this shifts in the volatile profile is consistent with the overall improvement in the aromatic quality of tobacco leaves achieved under the combined microbial-enzyme treatment. Consequently, these transformations likely contributed to the development of a more intense and persistent fruity and sweet aroma in the tobacco leaves (Zhang et al. 2025a). Figure 5B showed that the total volatile content in tobacco leaves varied considerably among the three aging treatments (*p* < 0.05). The control group (CK) exhibited aroma compound levels ranging from 124.74 to 142.95 μg/g. In contrast, the FJ treatments showed a notable increase, with total volatile contents reaching 283.21–300.78 μg/g, while the bacterial-enzyme co-treatment (JM) further enhanced accumulation, yielding concentrations between 317.06 and 388.80 μg/g. These results suggested that exogenous microbial inoculation promotes the conversion of flavor precursors, and that the microbial-enzyme co-treatment exerted a compounded effect on the synthesis of aroma compounds.

**Table 1:** Contents and odor activity values of the main aroma compounds in tobacco leaves with different aging treatments

	Compound	Odor description^a^	Content (μg/g)									Odor threshold (μg/g)^b^	Odor activity value								
			CK-3	CK-6	CK-9	FJ-3	FJ-6	FJ-9	JM-3	JM-6	JM-9		CK-3	CK-6	CK-9	FJ-3	FJ-6	FJ-9	JM-3	JM-6	JM-9
Esters	Dihydroactinidiolide	Sweet, creamy	2.18 ± 0.06d	0.02 ± 0.01i	0.3 ± 0.01 h	1.97 ± 0.04e	1.36 ± 0.02f	2.86 ± 0.04b	2.61 ± 0.06c	0.91 ± 0.07 g	5.42 ± 0.19a	0.5	4.36	< 1	0.60	3.94	2.72	5.72	5.22	1.82	10.84
	Ethyl myristate	Ester aroma, fruity	–	0.57 ± 0.04 g	0.47 ± 0.04 g	6.08 ± 0.03b	2.68 ± 0.13f	3.24 ± 0.08e	8.96 ± 0.08a	5.74 ± 0.1c	4.4 ± 0.11d	20	–	< 1	< 1	< 1	< 1	< 1	< 1	< 1	< 1
	Methyl palmitate	Iris scent0	–	–	–	5.06 ± 0.04d	–	14.61 ± 0.1b	9.38 ± 0.33c	–	22.09 ± 0.4a	4000	–	–	–	< 1	–	< 1	< 1	–	< 1
	Ethyl palmitate	Buttery	–	12.81 ± 0.12 h	15.69 ± 0.15 g	35.32 ± 0.92f	45.47 ± 0.65e	49.02 ± 0.33d	54.37 ± 1.28c	89.81 ± 0.96a	73.45 ± 0.72b	1.5	–	8.54	10.46	23.55	30.31	32.68	36.25	59.87	48.97
	Methyl linolenate	Melon flavor	–	2.91 ± 0.07f	6.83 ± 0.05d	–	7.27 ± 0.06d	8.65 ± 0.14c	–	18.03 ± 0.17a	9.47 ± 0.16b	/	–	–	–	–	–	–	–	–	–
	Ethyl linoleate	Fruity, floral	–	2.94 ± 0.08 g	5.55 ± 0.08f	14.62 ± 0.3e	15.97 ± 0.47d	14.27 ± 0.32e	27.23 ± 0.29b	36.65 ± 0.77a	21.41 ± 0.36c	2.8	–	1.05	1.98	5.22	5.70	5.10	9.73	13.09	7.65
	Ethyl linolenate	Waxy	–	5.14 ± 0.07 g	9.04 ± 0.13f	21.18 ± 0.45e	27.43 ± 0.41d	28.03 ± 0.17d	34.49 ± 0.66c	51.91 ± 0.81a	35.92 ± 0.76b	/	–	–	–	–	–	–	–	–	–
	Ethyl stearate	Wax aroma	–	0.41 ± 0.02 h	0.76 ± 0.04 g	5.29 ± 0.06c	2.96 ± 0.07f	4.47 ± 0.09e	9.46 ± 0.05a	6.59 ± 0.06b	5.07 ± 0.03d	18	–	< 1	< 1	< 1	< 1	< 1	< 1	< 1	< 1
	Dioctyl phthalate	Apricot flavor	–	–	3.07 ± 0.04d	5.4 ± 0.03c	0.17 ± 0.03e	0.52 ± 0.03e	13.69 ± 0.21b	–	18.42 ± 0.46a	/	–	–	–	–	–	–	–	–	–
	Methyl linoleate	Milky	–	0.85 ± 0.02e	–	16.13 ± 0.2b	21.93 ± 0.88a	–	9.14 ± 0.08c	7.43 ± 0.15d	–	/	–	–	–	–	–	–	–	–	–
Alcohols	Benzyl alcohol	Floral and fruity	12.44 ± 0.09d	7.38 ± 0.04f	2.92 ± 0.06 g	15.12 ± 0.4b	13.45 ± 0.09c	10.17 ± 0.29e	17.64 ± 0.18a	2.55 ± 0.11 g	12.82 ± 0.18d	10	1.24	< 1	< 1	1.51	1.35	1.02	1.76	0.26	1.28
	Linalool	Floral, sweet	2.54 ± 0.08d	4.19 ± 0.06b	0.32 ± 0.02f	3.38 ± 0.07c	3.23 ± 0.23c	2.59 ± 0.05d	3.93 ± 0.06b	5.58 ± 0.28a	0.62 ± 0.06e	0.037	68.65	113.24	8.65	91.35	87.30	70.00	106.22	150.81	16.76
	Phenethyl alcohol	Rose fragrance	9.35 ± 0.05e	–	3.89 ± 0.05f	20.27 ± 0.51b	20.03 ± 0.58b	–	25.1 ± 0.54a	17.94 ± 0.33c	14.96 ± 0.1d	31	< 1	–	< 1	< 1	< 1	–	< 1	< 1	< 1
	Furfuryl alcohol	Sweet aroma and caramel	1.14 ± 0.06c	–	0.26 ± 0.02d	3.39 ± 0.02b	–	–	4.3 ± 0.07a	–	–	0.07	16.29	–	3.71	48.43	–	–	61.43	–	–
Aldehydes	3-Furfural 3-Furaldehyde	Almond-like aroma	–	–	–	–	–	8.53 ± 0.26b	–	–	14.71 ± 0.13a	/	–	–	–	–	–	–	–	–	–
	Benzaldehyde	Almond flavor	0.93 ± 0.02e	–	0.11 ± 0.01f	2.34 ± 0.07b	0.05 ± 0.03f	1.37 ± 0.04d	2.14 ± 0.06c	0.01 ± 0f	3.25 ± 0.11a	0.35	2.66	–	< 1	6.69	< 1	3.91	6.11	< 1	9.29
	Phenylacetaldehyde	Fruity, nutty	6.87 ± 0.04b	4.35 ± 0.07d	–	–	–	4.76 ± 0.07c	–	–	8.44 ± 0.18a	1.8	3.82	2.42	–	–	–	2.64	–	–	4.69
	β-Cyclocitral	Floral and fruity fragrance	0.82 ± 0.02c	0.42 ± 0.02e	–	0.67 ± 0.06d	0.3 ± 0.02f	1.03 ± 0.03b	0.97 ± 0.08b	0.47 ± 0.03e	1.26 ± 0.04a	0.5	1.64	0.84	–	1.34	< 1	2.06	1.94	< 1	2.52
	Furfural	Caramel aroma	–	–	–	–	5.11 ± 0.08b	–	–	11.87 ± 0.04a	–	11.36	–	–	–	–	< 1	–	–	1.04	–
Ketones	Damascenone	Honey and floral notes	18.68 ± 0.12d	21.42 ± 0.03c	22.78 ± 0.25b	18.36 ± 0.68d	22.32 ± 0.56b	22.63 ± 0.19b	–	13.41 ± 0.18e	25.39 ± 0.63a	50	< 1	< 1	< 1	< 1	< 1	< 1	–	< 1	< 1
	Irisone	Floral and sweet	–	–	–	–	–	3.18 ± 0.06c	5.16 ± 0.06b	7.49 ± 0.19a	5.1 ± 0.02b	7	–	–	–	–	–	< 1	< 1	1.07	< 1
	4,7,9-Megastigmatrien-3-one	Sweet aroma of licorice	55.09 ± 0.26f	50.73 ± 0.19 g	60.82 ± 0.35e	87.88 ± 1.05b	87.4 ± 0.83b	86.53 ± 0.21b	65.71 ± 0.81d	68.21 ± 0.49c	92.42 ± 0.66a	/	–	–	–	–	–	–	–	–	–
	Phytone	Herbal scent	–	3.33 ± 0.09d	2.52 ± 0.1e	2.49 ± 0.04e	2.61 ± 0.08e	3.95 ± 0.1c	–	5.62 ± 0.26b	6.36 ± 0.12a	/	–	–	–	–	–	–	–	–	–
	Geranylacetone	Floral and fruity aroma	5.09 ± 0.06e	7.48 ± 0.04a	5.63 ± 0.08d	4.99 ± 0.04e	6.29 ± 0.17c	7.42 ± 0.11a	6.53 ± 0.22b	6.13 ± 0.09c	–	0.06	84.83	124.67	93.83	83.17	104.83	123.67	108.83	102.17	–
	α-Damascenone	Rose fragrance	–	3.58 ± 0.02c	0.41 ± 0.02d	–	7.44 ± 0.31a	0.55 ± 0.02d	–	4.96 ± 0.08b	–	0.013	–	275.38	31.54	–	572.31	42.31	–	381.54	–
	β-Ionone	Violet scent	–	3.04 ± 0.05c	0.69 ± 0.02d	3.95 ± 0.07b	4.6 ± 0.06a	–	–	–	–	0.0009	–	3377.78	766.67	4388.89	5111.11	–	–	–	–
Others	2-Methoxy-4-vinylphenol	Cedar and roasted peanut notes	8.38 ± 0.06c	–	0.89 ± 0.05e	10.26 ± 0.23b	2.71 ± 0.05d	–	15.38 ± 0.58a	8.45 ± 0.1c	–	/	–	–	–	–	–	–	–	–	–
	2-Acetylpyrrole	Nutty aroma	–	–	–	–	–	3.48 ± 0.03b	–	–	7.82 ± 0.16a	0.002	–	–	–	–	–	1740.00	–	–	3910.00
	2-Pentylfuran	Green bean and fruity notes	1.23 ± 0.09b	–	–	0.64 ± 0.04d	–	1.35 ± 0.02a	0.87 ± 0.02c	–	–	0.002	615.00	–	–	320.00	–	675.00	435.00	–	–

^a^Odor descriptions were obtained from the publicly available flavor database www.vcf-online.nl/VcfHome.cfm

^b^Odor thresholds were referenced from the online database www.vcf-online.nl/VcfHome.cfm and the compilation by (Van Gemert 2011), with all values corresponding to thresholds determined in air

CK: sterile water control; FJ: bacterial treatment; JM: bacterial-enzyme treatment

Figure 5C showed the heat map generated by studying the relative content of identified main aromatic compounds and their relationship with the properties of different aging methods of tobacco leaves. A total of 29 aromatic compounds were found in all the tobacco leaves, but they varied depending on the aging methods and aging time-point. The difference of volatile substances in different aging stages of tobacco leaves might be caused by the volatile precursor catabolic reactions (Zhu et al. 2025).

Volatile ketones, which arise from pathways such as β-oxidation and degradation of fatty acids, oxidative cleavage of carotenoids, and Maillard reactions, represented the most abundant class of aroma compounds in the analyzed samples and were characterized by their relatively low sensory thresholds (Wang et al. 2024b; Wu et al. 2023). The total ketone content in FJ-treated samples exceeded that of both CK and JM groups across all fermentation periods. Among the ketones identified, damascenone, 4,7,9-megastigmatrien-3-one, geranylacetone were the most abundant in all three tobacco types. Their concentrations increased progressively with aging in both FJ and JM samples, promoting the development of an ideal honey, floral, and fruity aroma in the tobacco leaves. These findings are consistent with previous studies reporting similar trends in other tobacco varieties (Wu et al. 2022). Shan et al. reported that the content of megastigmatrienone significantly increased in *B. velezensis* TB-1 fermented low-grade tobacco leaves (Shan et al. 2025).

Esters in tobacco leaves were primarily formed through esterification reactions between fatty acids and alcohols (Zhang et al. 2024c). The predominant volatile esters identified in aged tobacco leaves included methyl palmitate (35.32–89.81 μg/g), ethyl linoleate (21.18–51.91 μg/g), and ethyl linoleate (14.62–36.65 μg/g). Compared with the CK treatment, both the diversity and concentration of esters were higher in the FJ and JM treatments. This increase may be associated with the action of cellulase, which can degrade tobacco cell walls and thereby release additional precursors such as sugars and phenolic compounds. Concurrently, *B. clausii* likely promoted the transformation of these precursors into ester compounds through its oxidative metabolic pathways. Moreover, at the same aging time, the contents of most esters were higher in the JM treatment than that in the FJ treatment. As most esters contribute pleasant fruity or wine-like aromas (Wu et al. 2022), these changes in volatile composition are consistent with a positive effect of aging on tobacco flavor and with an overall improvement in tobacco quality through the enrichment of aroma-active compounds.

Alcohols in tobacco leaves originate primarily from the enzymatic oxidation and breakdown of polyunsaturated fatty acids, as well as from the secondary degradation of fatty acid hydroperoxides or microbial fermentation of carbohydrates (Weng et al. 2024). Under all three aging methods, the total alcohol concentration exhibited a gradual decline as fermentation progressed. Although alcohols generally contributed pleasant aromas and are known to mellow the smoke and reduce irritation (Zhang et al. 2025b), their overall abundance decreased over time. Similar result was also found in tobacco leaves fermented by *B. velezensis* TB-1, which reported that the content of 1,3-dioxolane-2-methanol and benzyl alcohol decreased (Shan et al. 2025). These findings aligned with previously reported trends in fermented tobacco leaves and further demonstrated that exogenous microbial-enzyme co-fermentation enhances ester formation (Zhang et al. 2025b). Notably, the concentrations of specific key alcohols varied significantly among treatments. The content of benzyl alcohol increased from 12.44 μg/g (CK) to 15.12 μg/g (FJ) and 17.64 μg/g (JM) for aging 3 months. A more pronounced increase was observed for furfuryl alcohol, which rose from 1.14 μg/g (CK) to 3.39 μg/g (FJ) and 4.30 μg/g (JM). Benzyl alcohol, derived from the conversion of phenylalanine, serves as an important odor-active compound in tobacco, contributing distinct aromatic characteristics to cigarette smoke (Weng et al. 2024). Furfuryl alcohol imparted a sweet, caramel-like scent that significantly enriched the overall aroma (Liu et al. 2024). Furthermore, it played an effective role in reducing smoke irritation and masking undesirable odors in the tobacco leaves.

A total of only five volatile aldehydes were identified across the three types of aged tobacco leaves. Aldehydes—mainly derived from lipid oxidation—are known for their low odor thresholds and play a significant role in shaping the aroma profile of tobacco leaves (Fang et al. 2024). As shown in Fig. 5B and 5C, the total aldehyde content under the JM treatment was significantly higher than that in both the CK and FJ treatments at each aging time point. Moreover, aldehyde levels increased progressively throughout the fermentation period, suggesting that microbial-enzyme co-fermentation promoted the degradation and conversion of lipid, leading to increased aldehyde formation (Li et al. 2024). The five aldehydes detected during aging included 3-furaldehyde, benzaldehyde, phenylacetaldehyde, β-cyclocitral, and furfural. In all treatments, aldehydes collectively accounted for less than 7.5% of the total quantified aroma compounds. Their concentrations increased linearly over fermentation time in both FJ and JM samples. At 9-month aging stage, three aldehydes—3-furraldehyde, phenylacetaldehyde, and β-cyclocitral—were detected only in the FJ and JM groups, and their concentrations were higher in the JM than in FJ, while they were not detected in CK group. In contrast, benzaldehyde was detected in all three treatments; its content was highest in JM (3.25 μg/g), followed by FJ (1.37 μg/g) and CK (0.11 μg/g). These aldehydes contribute fruity and nutty notes, substantially enriching the complexity and layering of the overall tobacco aroma.

The OAVs of key aroma components in tobacco leaves subjected to different treatments are summarized in Table 1. The results indicated that the JM treatment exhibited significantly higher OAVs for dihydroactinidiolide and ethyl palmitate—both associated with milky notes—compared to the CK and FJ treatments. With OAVs exceeding 1, these compounds contributed to a more pronounced milky aroma in the JM group. Similarly, the JM treatment exhibited higher OAVs for pleasant aroma compounds, including ethyl linoleate (fruity and floral), linalool (sweet, floral), benzaldehyde (almond scent), β-ionone (violet scent), benzyl alcohol (fruity and floral), and β-cyclocitral (fruity and floral). Among these, the OAVs of ethyl linoleate, linalool, and benzaldehyde were greater than 1, enhancing the fruity aroma profile of the JM treatment. Additionally, furfuryl alcohol, which imparted a caramel-like sweetness, displayed an OAV above 1 in the JM treatment, underscoring its role in enriching the sweet aromatic notes. Notably, benzaldehyde and 2-acetylpyrrole (nutty aromas) also exhibited higher OAVs in the JM treatment. Owing to its particularly low odor threshold, 2-acetylpyrrole yielded the highest OAV among all detected aroma compounds. Collectively, these substances, including ethyl linoleate, benzyl alcohol, linalool, benzaldehyde, phenylacetaldehyde, β-cyclocitral, β-ionone, and 2-acetylpyrrole, contributed significantly to the overall aroma profile of the JM treatment. With the exception of benzaldehyde, β-cyclocitral, and β-ionone, all exhibited OAVs greater than 1, identifying them as key aroma-active compounds following tobacco fermentation.

### Characteristic aroma compounds in tobacco leaves with different aging treatments

**Fig. 6:**
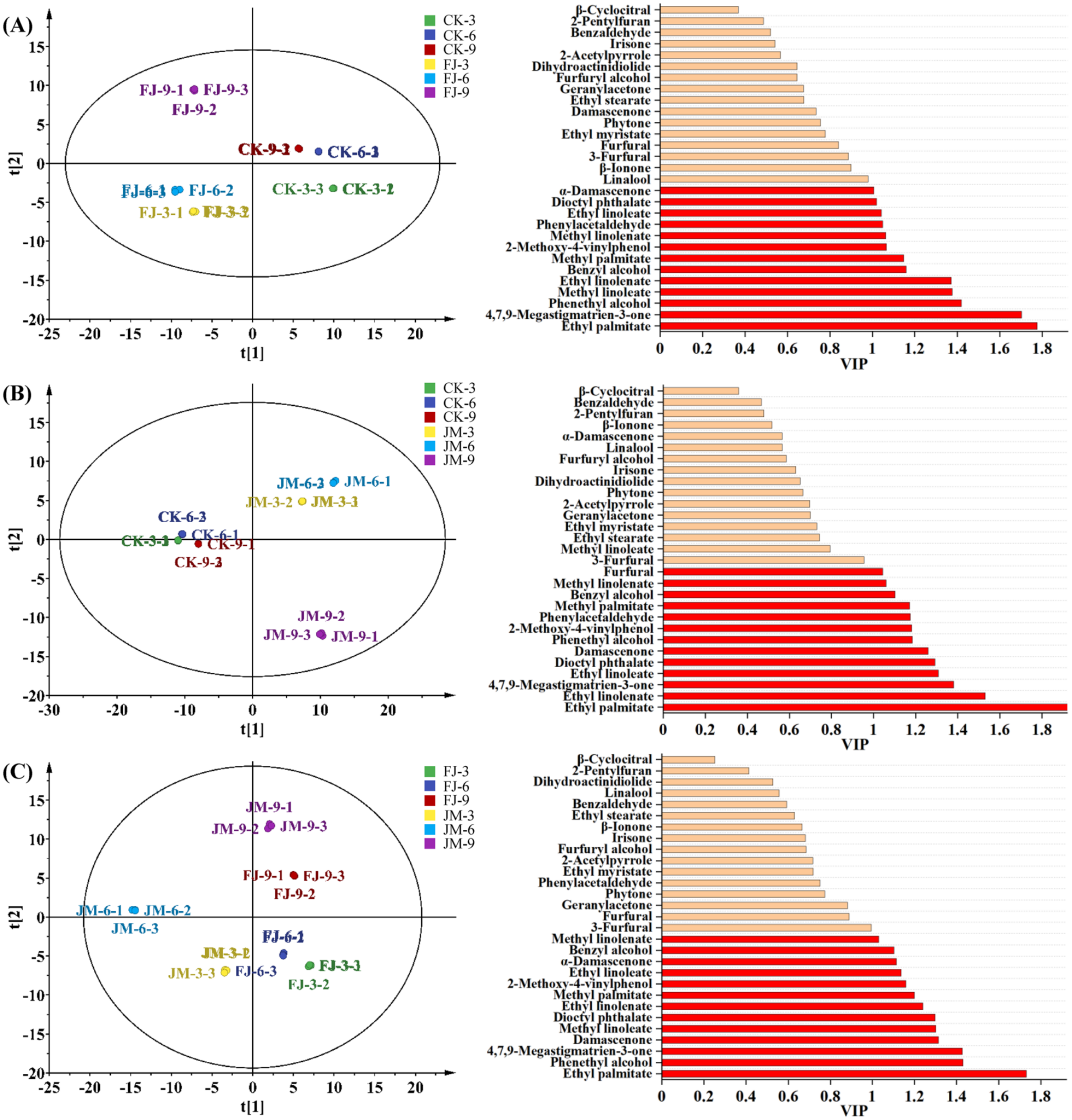
PLS-DA score plot and VIP values of volatile compounds in tobacco leaves with different aging treatments. **A** CK vs FJ, Rx^2^ is 0.998, Ry^2^ is 0.999, and Q^2^ is 0.998; **B** CK vs JM, Rx^2^ is 0.982, Ry^2^ is 0.800, and Q^2^ is 0.991; **C** FJ vs JM, Rx^2^ is 0.998, Ry^2^ is 0.998, and Q^2^ is 0.996. CK: sterile water control; FJ: bacterial treatment; JM: bacterial-enzyme treatment

A PLS-DA model was applied to analyze the aroma compounds in tobacco leaves subjected to different aging treatments, as depicted in Fig. 6. The model exhibited high reliability, with both the variable fit index (R^2^) and the predictive ability index (Q^2^) exceeding 0.5, indicating strong explanatory power and predictive ability. Variables with a variable importance in projection (VIP) > 1 and *p* < 0.05 were considered as differential aroma compounds (Wang et al. 2024a). When comparing the FJ group to the CK group, key differential compounds included ethyl palmitate, 4,7,9-megastigmatrien-3-one, phenethyl alcohol, methyl linoleate, ethyl linolenate, benzyl alcohol, and methyl palmitate. In the JM group versus CK, the main differential volatiles were ethyl palmitate, ethyl linolenate, 4,7,9-megastigmatrien-3-one, ethyl linoleate, dioctyl phthalate, and damascenone. Among these, 13 core characteristic compounds were consistently identified across at least two pairwise comparisons: ethyl palmitate, 4,7,9-megastigmatrien-3-one, phenethyl alcohol, methyl linoleate, ethyl linoleate, benzyl alcohol, methyl linolenate, α-damascenone, damascenone, 2-methoxy-4-vinylphenol, ethyl linoleate, phenylacetaldehyde, and dioctyl phthalate. These substances were major contributors to the sensory differences among treatments and associated with desirable aroma attributes such as fruity, floral, and creamy notes (Table 1).

### Correlation analysis between dominant microorganisms and characteristic aroma compounds

**Fig. 7:**
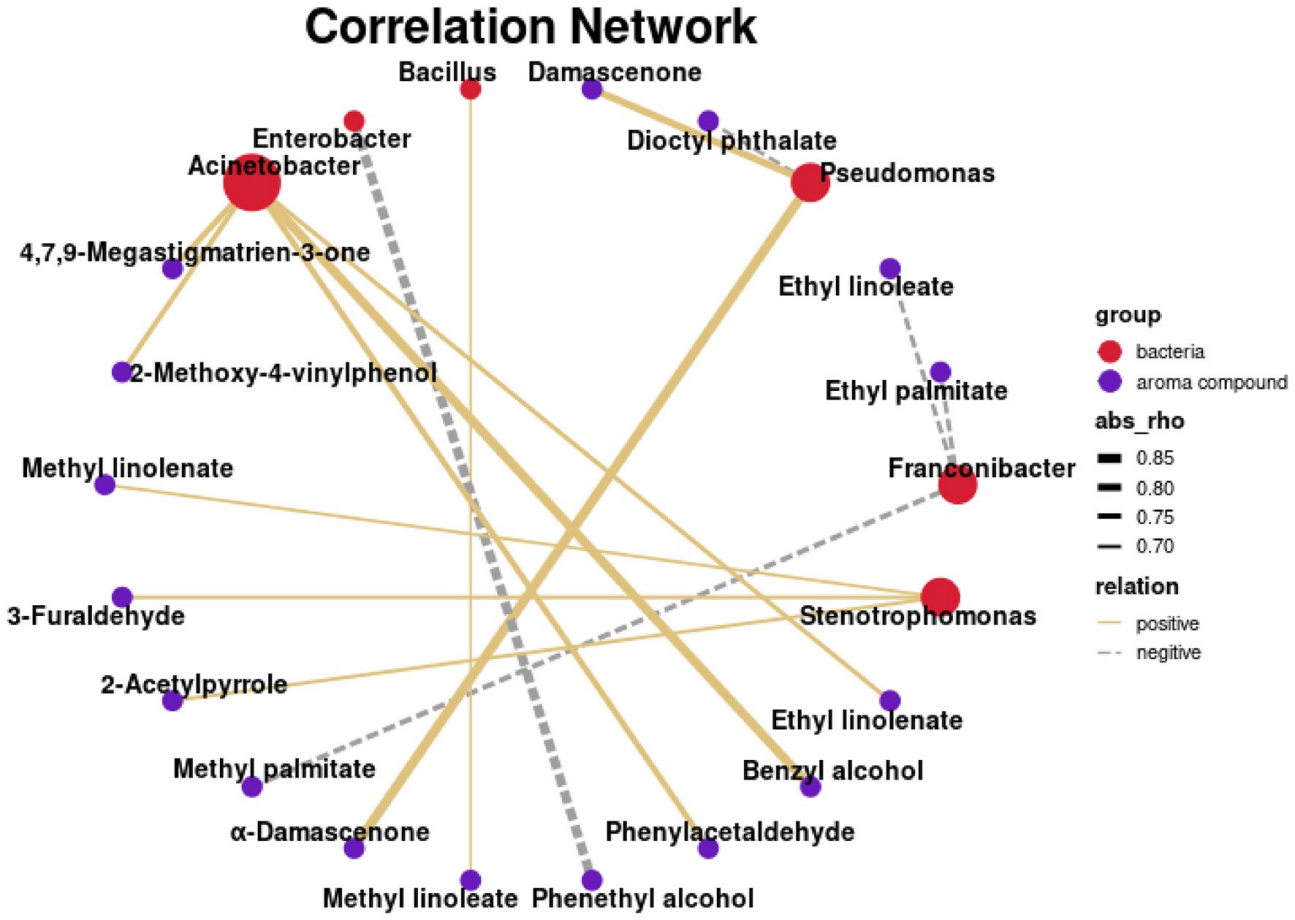
Correlation network between dominant bacteria and characteristic aroma compounds identified in tobacco leaves with different aging treatments. Red nodes represent bacteria, while blue nodes represent aroma compound. Edges represent significant Spearman correlations (|ρ|> 0.7, *p* < 0.05)

To elucidate the role of dominant microbiota in flavor formation, which is likely mediated by their high metabolic potential, this study investigated the Spearman’s correlations between the top ten bacterial genera and 13 characteristic aroma components (VIP > 1, *p* < 0.05). Significant correlations were defined using a threshold of |ρ|> 0.7 and *p* < 0.05. Spearman correlation analysis and visualized using correlation networks (Fig. 7). Results showed that *Pseudomonas* exhibited correlations with three aroma compounds, showing positive correlations with α-damascenone, damascenone, but a negative correlation with dioctyl phthalate, while *Stenotrophomonas* was positively correlated with 3-furaldehyde, 2-acetylpyrrole, and methyl linolenate. Previous study has implicated *Pseudomonas* and *Stenotrophomonas* in the formation of tobacco flavor, showing a significant positive correlation with compounds such as acetophenone, decyl aldehyde, and β-cyclonitroaldehyde during industrial fermentation, thereby highlighting their potential contribution to the sensory profile (Zhang et al. 2024b). *Franconibacter* was exclusively negatively correlated with ethyl palmitate, ethyl linoleate, and methyl palmitate, indicating a potential inhibitory or competitive role in the formation of these aroma-related metabolites. *Enterobacter* displayed a specific negative correlation only with phenethyl alcohol, whereas *Bacillus* showed a single positive correlation with methyl linoleate. In contrast, this pattern differs from the findings of Zhang et al., who identified that *Solibacillus* as a characteristic microorganism during the air-curing process and reported a significant positive correlation between its abundance and the formation of carbonyl compounds such as 3,5-octadien-2-one, geranyl acetone, and 2,3-pentanedione (Zhang et al. 2025a). The limited number of direct associations observed for *Bacillus* implied that its contributions to the fermentation process was likely indirect, acting primarily through the modulation of substrate availability and microbial community structure rather than through direct aroma biosynthesis. Collectively, these findings preliminarily elucidate the potential contribution of bacterial communities to aroma compound formation during tobacco leaf aging. Given that bacterial communities have been shown to exhibit stronger associations with variations in aroma compounds during fermentation (Zhang et al. 2024b), this study focused primarily on bacterial community dynamics to establish a foundational understanding of their role in flavor development. Nevertheless, 16S rRNA sequencing provides only genus-level resolution and does not enable strain-level discrimination within complex taxa such as *Bacillus*. In addition, fungal communities were not analyzed in this study. Future work integrating strain-specific tracking and fungal community profiling will be necessary to obtain a more comprehensive understanding of the microbial mechanisms underlying flavor formation.

## Conclusion

This study systematically evaluated the impact of bacterial and bacterial-enzyme co-treatments on the microbial community structure and aroma profile of tobacco leaves during aging. High-throughput sequencing demonstrated that the application of JM treatment significantly enhanced microbial diversity, promoted the proliferation of beneficial phyla such as Bacteroidota and Cyanobacteria. Functional prediction via PICRUSt highlighted the dominance of metabolic pathways—especially carbohydrate metabolism, amino acid metabolism, and metabolism of cofactors and vitamins—which are closely associated with the formation of key aroma compounds. Volatile compound analysis revealed that the JM notably increased the total content of aroma substances, particularly esters and ketones, while facilitating the conversion of alcohols into more desirable aromatic compounds. Key differential volatiles, including ethyl palmitate, ethyl linolenate, α-damascenone, and damascenone, were identified as characteristic markers in JM, contributing to enhanced fruity, floral, and milky notes. Odor activity value analysis further confirmed the sensory significance of compounds such as dihydroactinidiolide, ethyl linoleate, linalool, and 2-acetylpyrrole in JM. Correlation network analysis elucidated the relationships between dominant microbial genera and characteristic aroma components. *Acinetobacter*, *Pseudomonas*, and *Bacillus* were positively correlated with multiple key volatiles, underscoring their roles in flavor formation. In summary, microbial-enzyme co-fermentation significantly modulated the microbial community structure and metabolic potential of tobacco leaves, thereby enhancing the accumulation of desirable aroma compounds and improving overall aroma quality. Although 16S rRNA-based functional prediction has inherent limitations, the present results provide robust evidence that combined microbial–enzyme treatments are an effective strategy for accelerating aging and improving tobacco quality. Future studies based on metagenomics and functional validation of key strains will further refine the mechanistic understanding. This study provides a theoretical foundation and a practical guidance for the industrial application of directed fermentation, paving the way for standardized production of high-quality aged tobacco.

## Supplementary Information

Additional file1 (DOCX 374 KB)

## Data Availability

Data will be made available on request.
