# Supplementary material for: Microbial-enzyme synergistic treatment stabilizes surface microbial communities and enhances flavor quality during tobacco leaf aging
Source: Bioresour Bioprocess. 2026 Apr 15;13(1):53. doi: 10.1186/s40643-026-01052-1 (PMC13079249; doi:10.1186/s40643-026-01052-1)
Supplement: Supplementary file 1 — Additional file1 (DOCX 374 KB) [file 40643_2026_1052_MOESM1_ESM.docx]

**Microbial-enzyme synergistic treatment stabilizes surface microbial communities and enhances flavor quality during tobacco leaf aging**

Xu Chunping^1^, Sun Yizhe^1^, Fan Yuntao^2^, Qu Lili^1^, Xiao Zhang^3^, Ding Meizhou^4*^, Ma Rong^2*^

1. College of Tobacco Science and Engineering, Zhengzhou University of Light Industry, Zhengzhou 450000, China;

2. Research Center, Hebei Tobacco Industry Co., Ltd., Shijiazhuang 052165, China;

3. Henan High-Tech Industry Co., Ltd., Henan Academy of Sciences, Zhengzhou 450000, China;

4. Research Center, Henan Tobacco Industry Co., Ltd., Zhengzhou, 450000, China

Correspondence: Ma Rong (15286811817@163.com) and Ding Meizhou (dingmeizhou999@163.com)

**Supplementary Materials**

**Figure S1**. Rarefaction curves of bacterial communities in tobacco leaves with different aging treatments. The x-axis indicates the number of sequences sampled, and the y-axis represents the corresponding number of observed OTUs. CK: sterile water control; FJ: bacterial treatment; JM: bacterial-enzyme treatment.


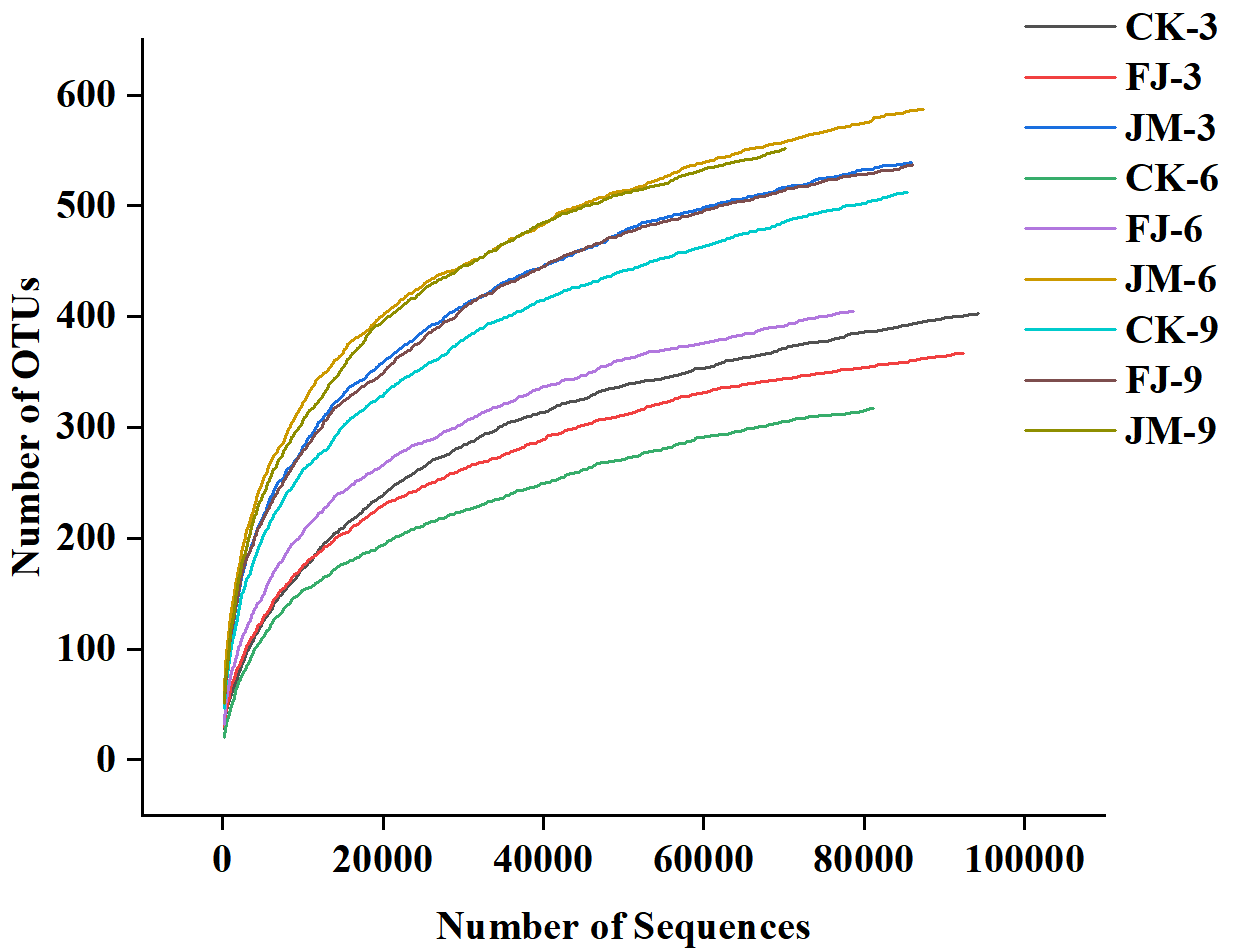


**Figure S2**. Linear discriminant analysis (LDA) score of LEfSe in tobacco leaves with different aging treatments. (A) CK; (B) FJ; (C) JM. CK: sterile water control; FJ: bacterial treatment; JM: bacterial-enzyme treatment.


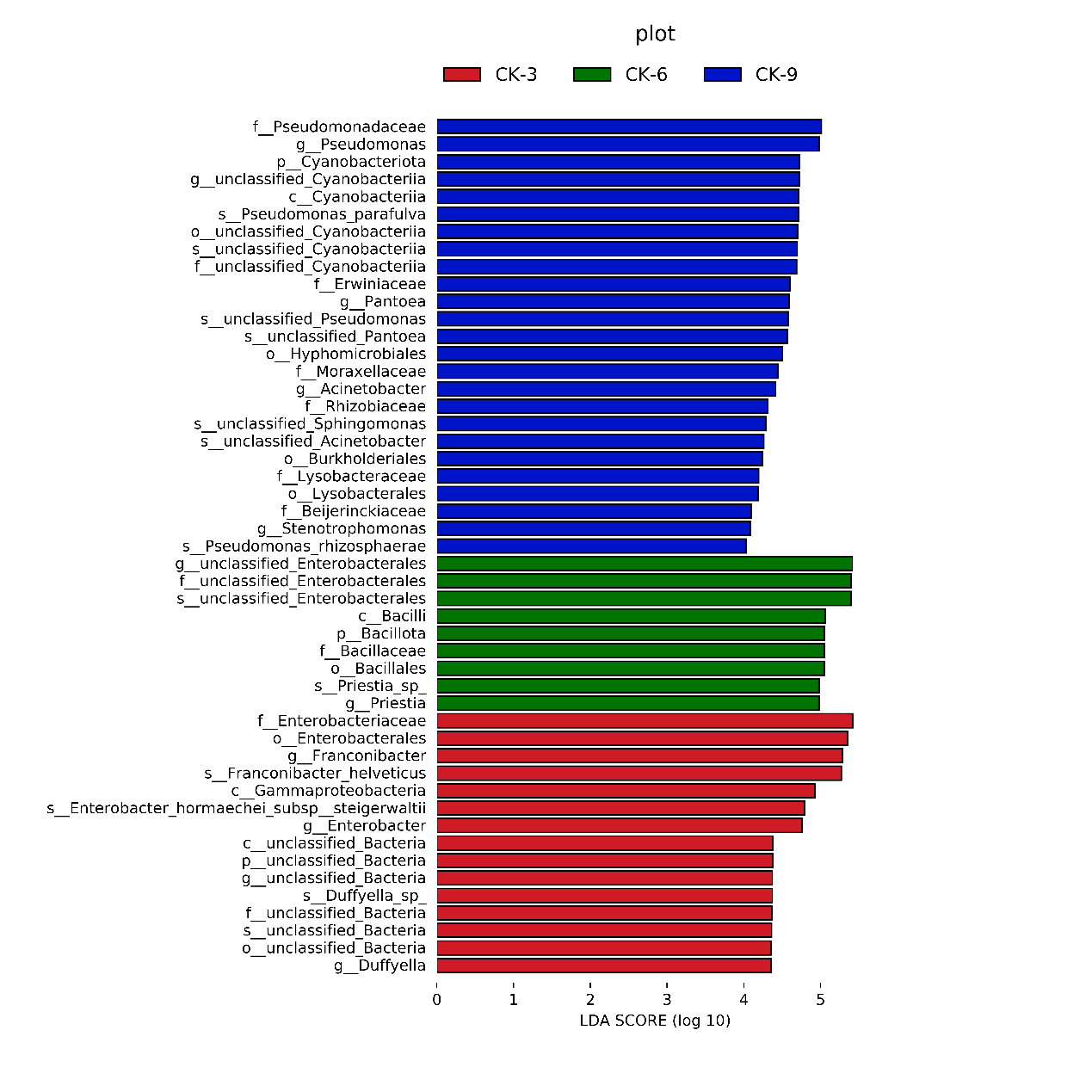


(B)

**(B)**

(A)


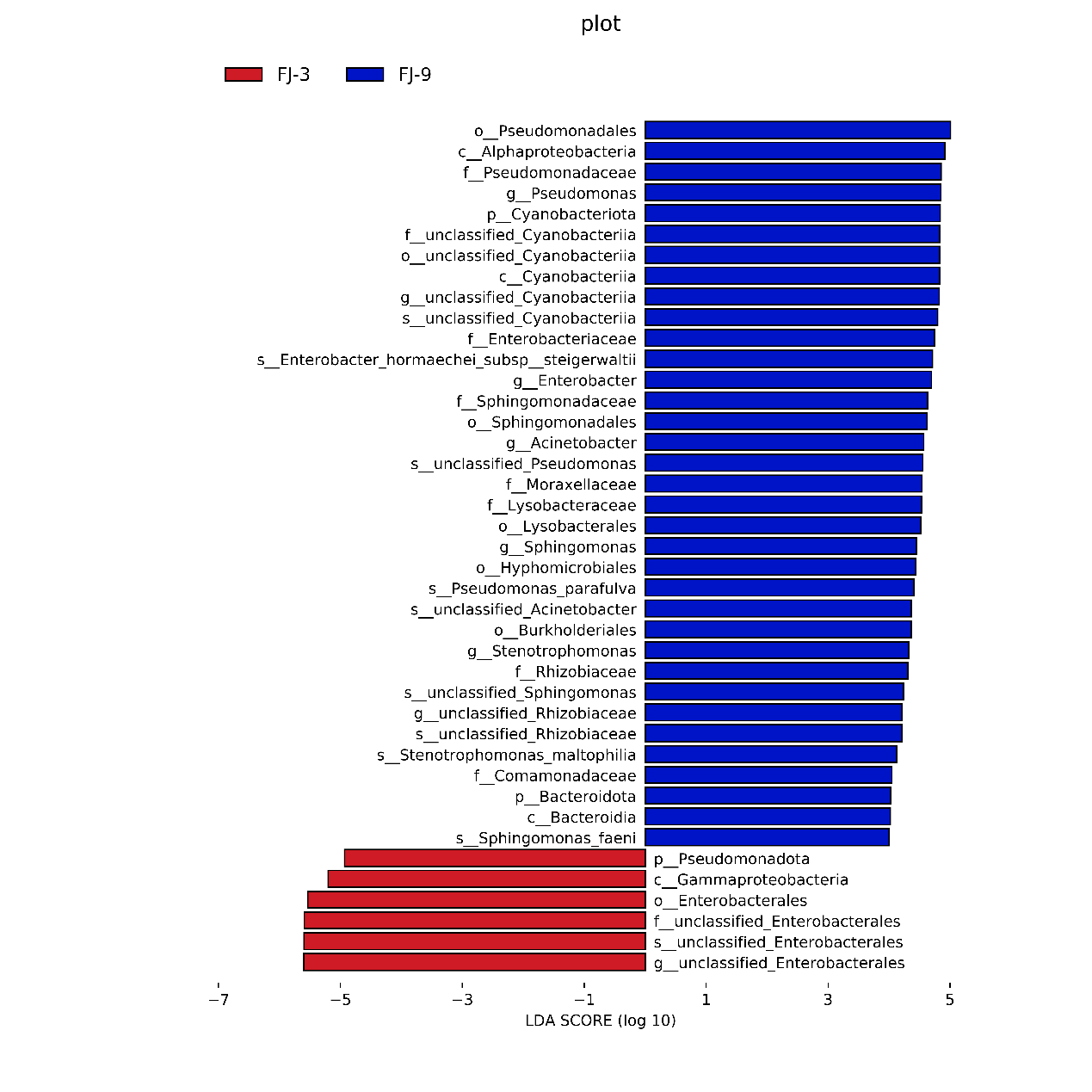


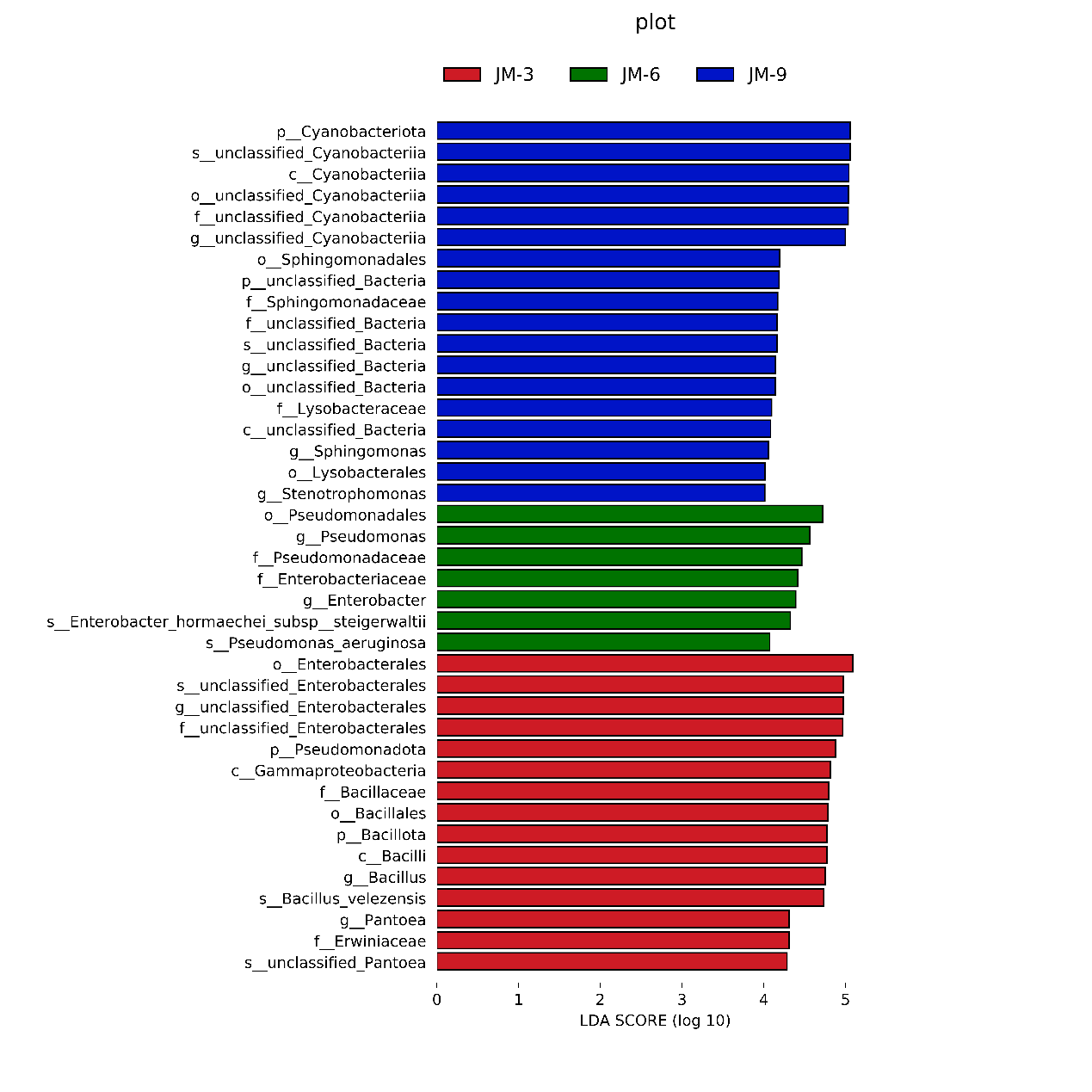


(C)

**Table S1.** Coverage of bacterial sequence reads after removing singletons in tobacco leaves with different aging treatments. CK: sterile water control; FJ: bacterial treatment; JM: bacterial-enzyme treatment.

| Sample ID | Number | OTUs | Coverage |
| --- | --- | --- | --- |
| CK-3 | 94247 | 403 | 0.999 |
| FJ-3 | 92448 | 367 | 0.999 |
| JM-3 | 85941 | 539 | 0.999 |
| CK-6 | 81103 | 317 | 0.999 |
| FJ-6 | 78697 | 405 | 0.999 |
| JM-6 | 87463 | 587 | 0.999 |
| CK-9 | 85348 | 512 | 0.999 |
| FJ-9 | 86076 | 537 | 0.999 |
| JM-9 | 70182 | 552 | 0.998 |
